# The impact of morbid obesity on survival of endometrial cancer

**DOI:** 10.4274/tjod.galenos.2020.83773

**Published:** 2020-10-02

**Authors:** Ahmet Barış Güzel, Ghanim Khatib, Ümran Küçükgöz Güleç, Derya Gümürdülü, Mehmet Ali Vardar

**Affiliations:** 1 Çukurova University Faculty of Medicine, Department of Obstetrics and Gynecology, Adana, Turkey; 2 Çukurova University Faculty of Medicine, Department of Pathology, Division of Gynecologic Pathology, Adana, Turkey

**Keywords:** Morbid obesity, endometrial cancer, survival

## Abstract

**Objective:**

Morbid obesity is identified as patients with a body mass index more than 40 kg/m^2^. Obesity is known as a risk factor for endometrial cancer due to the increase of the deposited estrogen. This study was conducted to evaluate the effect of morbid obesity on the survival of endometrial cancer.

**Materials and Methods:**

The archival records and pathologic reports of patients with endometrial cancer who underwent surgery and were followed up in Çukurova University Gynecologic Oncology Center between January 1996 and December 2018 were reviewed, retrospectively. Data regarding body mass index and survival was reported in 520 patients. These patients were stratified into two groups according to their body mass index, <40 and ≥40 kg/m^2^. The groups’ clinic, pathologic features, and survival rates were compared.

**Results:**

There were 146 patients in the morbidly obese group and 374 patients in the obese group. The mean age of the groups was 58.5 and 56.2 years, respectively. The mean follow-up time was 51.6 months. Comorbidities were significantly higher in the morbidly obese group. The five-year disease-free and overall survival rates were 78.3% and 85.3% in the morbidly obese group, and 81.6% and 90.1% in the obese group, respectively. Although the groups’ clinical and pathologic features were homogeneously distributed, disease-free and overall survival rates were significantly different (p=0.053 and p=0.054, respectively).

**Conclusion:**

Morbidly obese patients with endometrial cancer were associated with 2.7-fold increased risk of death and 1.7-fold increased risk of recurrence compared with those who had body mass index <40 kg/m^2^. It is important to deal with the frequent comorbidities in this special group, which could be simply altered by lifestyle changes. Morbidly obese patients with endometrial cancer should be encouraged in lifestyle changes and consulted by dieticians and endocrinologists.

**PRECIS:** Although there are limited studies on the effect of obesity on long-term outcomes of endometrial cancer, morbid obesity has not been considered separately in most of them. We address this issue herein.

## Introduction

Endometrial carcinoma is the most seen gynecologic cancer in the developed world^(1)^. Based on epidemiologic and histologic evidence, endometrial cancer has been historically stratified into two types^(2)^. Endometrioid type (type 1), which comprises about 80% of the cases, is an unopposed estrogen-dependent endometrial cancer type^(2,3)^. It has been supposed that the estrogen deposited in the adipose tissue leaves the endometrium layer under continuous hormonal exposure^(4)^. Hence, the relation of obesity with endometrial cancer risk, particularly the endometrioid type, has been emphasized since the dualistic identification^(2,3,4,5)^. Therefore, the growing incidence of endometrial carcinoma in the last decades is presumably due to the rising obesity levels^(6)^. The World Health Organization (WHO) defined obesity as patients with body mass index (BMI) more than 30 kg/m^2^ and morbid obesity as ≥40 kg/m^2 (7)^. It has been reported that up to 81% of the endometrial cancer’ patients are obese and 19% to 36% of them are morbidly obese^(6)^. Inadequate activity and obesity-linked medical comorbidities in these patients were supposed to contribute in the management complexity and to negatively affect long-term outcomes^(6,8)^. Even though this special population is characterized with the low-grade, early-stage, and good prognostic endometrioid type endometrial cancers, their mortality seems to be higher compared with their normal weight (kg) counterparts^(5,8,9,10)^. However, the underlying reason and mechanism for this condition is uncertain. In other words, it is not clear whether obesity itself has a negative prognostic influence on endometrial cancer or its comorbidities that lead to such a result^(3,5)^. Furthermore, there are insufficient studies on the issue, focusing on morbidly obese patients (BMI ≥40 kg/m^2^) with endometrial cancer. In the present study, the impact of morbid obesity on the survival of endometrial cancer was investigated.

## Materials and Methods

The archival records and pathologic reports of patients with endometrial cancer who underwent surgery and were followed up in Çukurova University Gynecologic Oncology Center between January 1996 and December 2018, were reviewed, retrospectively. Data regarding BMI and survival was reached in 520 patients. These patients were stratified into two groups according to their BMI, <40 and ≥40. BMI [kg/height (m)]^2^ was calculated and classified regarding to the WHO guidelines. Thus, patients with BMI ≥40 kg/m^2^ were identified as morbidly obese. The groups’ clinic, pathologic features, and survival rates were compared. Compared variables included age, comorbidities, surgical approach, surgical procedure, perioperative and postoperative complications including wound infections, hospitalization time, tumor size, histologic type, stage, grade, myometrial invasion, retroperitoneal lymph node (LN) involvement, yielded LN count, lymphovascular space invasion, adjuvant treatments, and follow-up data.

This study was performed in accordance with the ethical standards of the Helsinki Declaration. Ethical approval was not obtained for this study because of its retrospective nature. A informed consent was obtained routinely.

The main surgical procedures were total hysterectomy-bilateral salpingo-oophorectomy (via laparotomy or laparoscopy) with or without pelvic and para-aortic lymphadenectomy according to the intraoperative frozen section result. Intraoperative frozen section was applied for all included cases, and a decision whether to pursue lymphadenectomy was taken based on its results. Lymphadenectomy was not performed in patients with stage 1a, International Federation of Gynecology and Obstetrics (FIGO) grade 1-2, and <2 cm tumors (low-risk factors). Lymphadenectomy (± omentectomy) was considered in the presence of any of the following: endometrioid adenocarcinoma grade 3, tumor diameter >2 cm, ≥50% myometrial invasion, stage >1a or non-endometrioid histologies. Adjuvant therapies (brachytherapy, external beam radiotherapy and/or chemotherapy) were kept in view for patients with ≥ intermediate risk factors. All specimens were assessed by gynecologic pathologists. Comorbidities were accepted as any concomitant chronic disease. The FIGO 2009 staging guideline for endometrial cancer was used^(11)^. Stage of cases operated before 2009 was rearranged according to this recent staging system. Grade was also identified according to the 1988 FIGO grading system^(11,12)^. The period between the date of the histopathologic diagnosis and recurrence was identified as disease-free survival (DFS). Overall survival (OS) was considered to be the time between the date of the histopathologic diagnosis and date of death from any cause.

### Statistical Analysis

Data were analyzed using the SPSS software version 23.0 (IBM, Armonk, NY, USA). Descriptive analyses are presented as mean ± standard deviation, number and percentage. Normally distributed continuous variables were analyzed using Student’s t-test. Categorical data were analyzed using the chi-square test or Fisher’s Exact test. Survival analysis was realized using the Kaplan-Meier method and the differences in the survival curves were calculated through the log-rank test. The significance of multiple variables was assessed using the Cox proportional hazard model. P-values were considered significant at the level <0.05.

## Results

The groups’ characteristics are summarized in Table 1. Unlike age, comorbidities and LN dissection, all clinical, surgical, and pathologic variables were identical between the groups. The mean age of the morbidly obese group and obese group was 58.5±10 years and 56.3±10.7 years, respectively (p=0.033). The rate of comorbidities was significantly higher in the morbidly obese group compared with the obese group (64.6% vs 53.2%, respectively, p=0.020).

More than half of the patients in both groups underwent laparoscopic surgery, 55.5% of the patients with morbid obesity and 54.8% obese group. No difference between the groups was observed with respect to the surgical approach (p=0.895). Wound infections were developed in 6.3% of the morbidly obese group and in 3.5% of the obese group. However, there was no significant difference between the groups according to the intraoperative and postoperative complications including wound infections. Pelvic and para-aortic lymphadenectomy was performed less frequently in the morbidly obese group compared with the obese group (17.8% vs 34.6%, respectively). However, it should be noted that comparable lymphadenectomy ratios of both groups were recorded when pelvic LN dissection was performed exclusively (9.6% and 10.5%, respectively).

Endometrioid type endometrial cancer was found in 81.5% of the morbidly obese group and 76.1% the obese group. With respect to the histopathologic type, no significant difference was determined between the groups (p=0.182). There was also no difference regarding to grade distribution between the groups (p=0.578). Most cases of both groups were confined to the uterus, 87% of the morbidly obese group and 85.4% of the obese group, without a significant difference (p=0.650). The myometrium was invaded less than 50% in 65.9% and 69.7% of the morbidly obese group and obese group, respectively. No significantly difference was detected between the groups in terms of myometrial invasion (p=0.416). The ratio of lymphovascular space invasion was also not significantly different between the groups; 34.7% in the morbidly obese group and 31.8% in the obese group. The involved LN rate was 6.1% in the morbidly obese group and 10.1% in the obese group, with no significant difference (p=0.163). Furthermore, there was no significant difference between the groups concerning adjuvant treatments (p=0.922) (see Table 1).

**Figure 1:**
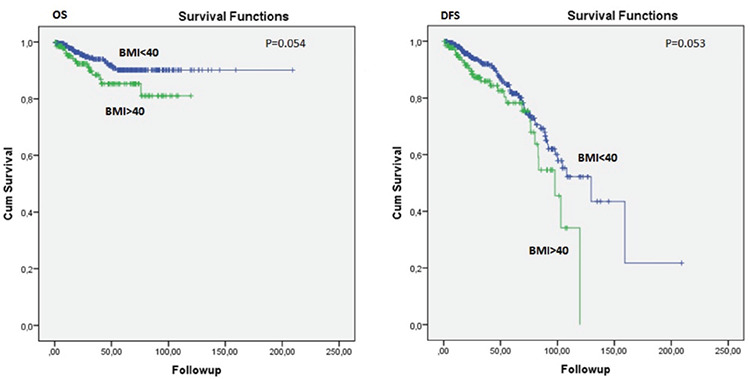
Survival curves of the groups DFS: Disease-free survival, OS: Overall survival, BMI: Body mass index

The mean follow-up period was 51 months. The 5-year OS of the morbidly obese group and obese group was 85.3% and 90.1%, respectively (p=0.054). The 5-year DFS of the morbidly obese group was 78.3% and 81.6% in the obese group, and this difference was relatively significant (p=0.053). The survival curves of the groups are demonstrated in Figure 1. Significant variables determined with the univariate analysis were assessed using a Cox regression hazard model (Table 2). Comorbidities, stage, and BMI were detected as independent prognostic factors for OS. For DFS, only myometrial invasion and BMI were found to be independent prognostic factors. Patients with endometrial cancer who were morbidly obese were associated with 2.7-fold (1.11-6.58; p=0.028) increased risk of death and 1.7-fold (1.02-3.07; p=0.042) increased risk of recurrence compared with those who had a BMI <40 kg/m^2^.

## Discussion

Obesity, and morbid obesity in particular, is a growing issue around the world. Obesity is a well-known predisposing factor for several metabolic diseases, as well as various malignancies^(4,13)^. It was reported that compared with the normal-kg population, patients with BMI >40 kg/m^2 ^were associated with a 60% higher risk of death from all cancers^(8,9)^. Additionally, obesity was considered as a risk factor for recurrence in various malignancies such as breast, colon, and prostate cancers^(14)^. The robust association between obesity and endometrial cancer risk has been emphasized in many studies^(3,4,14)^. It was reported that patients with morbid obesity had a 9-fold increased risk for endometrial cancer as compared with the normal-kg population^(4)^. However, even though there are limited studies in which the effect of obesity on the long-term outcomes of endometrial cancer were evaluated, morbid obesity was not taken into account separately in most of them. In the current study, the impact of morbid obesity on the survival of the endometrial cancer was exclusively investigated. Herein, we found a tendency toward lower DFS (p=0.053) and OS (p=0.054) in patients with morbid obesity compared with those with a BMI <40 kg/m^2^. Furthermore, morbid obesity was detected to be an independent prognostic factor for both DFS and OS.

In a prospective cohort study with more than 900,000 participants, the relative risk of death from endometrial cancer for patients with BMI 30-34 and >40 kg/m^2 ^was recorded as 2.53 and 6.25, respectively^(9)^. Arem and Irwin^(3)^ reported that worse survival was noticed in four studies included in their review, and risk was greatest (1.86-2.76) in women with morbid obesity. In a Gynecologic Oncology Group study, von Gruenigen et al.^(14)^ determined that obesity was related to an increased risk of mortality but not increased recurrence rates in patients with early-stage endometrial cancer. Also, the ancillary data analysis of the Gynecologic Oncology Group LAP2 study illustrated that obesity was linked to all-cause mortality but not cancer-specific mortality^(5)^. By contrast, in our study, morbidly obese patients with endometrial cancer were compared with obese patients with endometrial, not only with the normal-kg women with endometrial cancer. In other words, solely morbid obesity was the point of our study, likely leading to the difference with the abovementioned studies concerning DFS. In addition, all women, regardless of disease stage, not only patients with early-stage endometrial cancer, were included in our study.

Several arguments have been proposed to explain the rationale of the association between obesity and mortality in women with endometrial cancer^(15)^. Medical conditions such as hypertension, diabetes, and cardiovascular diseases, the increased surgical complexity, operation time, and blood loss were suggested as influencers in this relationship^(3,16,17,18)^. Moreover, physiologic alterations including chronic inflammation, insulin resistance, changes in lipid and hormone profiles in patients with obesity were offered as mediators for this association^(3,19)^. Conversely, some studies failed to demonstrate a relationship between obesity and endometrial cancer prognosis^(3,13)^. It would not be fair to attribute the worse survival in these patients to the obesity-linked tumor prognostic features because endometrial cancer tends to be with favorable grade and histology in obese women. However, it should be remembered that this association could harbor multiple confounders such as age, patient, and tumor characteristics^(15)^. Temkin et al.^(13)^ stated that BMI was not an independent prognosticator for survival of endometrial cancer, and the attributed favorable potency was because of younger age, low grade, and early stage of the tumor, not due to obesity itself. Furthermore, it was shown in some studies that BMI was not an independent predictor for endometrial cancer stage^(13,15,20)^. Therefore, LN evaluation should not be omitted thanks to the favorable-disease-argument in women with obesity. However, serious technical difficulties of lymphadenectomy in patients with morbid obesity should be kept in mind. In our study, pelvic lymphadenectomy was comparable between the groups, but para-aortic LN dissection was significantly lower in the morbidly obese group compared with the obese group. Despite the relative high rate of lymphadenectomy in both groups of our study, no difference between them was detected in terms of per- and postoperative complications, and these findings were commensurate with some literature studies, but not with others^(21)^.

Beyond the association between obesity and survival, there is robust evidence of increased quality of life of patients with endometrial cancer made through lifestyle alterations such as physical activity, kg, and diet^(3,22)^. Higher health-related quality of life was reported in patients who followed more dedicated lifestyle recommendations^(22,23,24)^.

### Study Limitations

The retrospective nature and its potential biases are the main weaknesses of our study. However, the limitation the study population to women with morbid obesity, the large cohort from a single academic cancer center, surgery and evaluation of all cases by the same team of gynecologic oncologists and gynecologic pathologists, and the long follow-up period were the main strengths.

## Conclusion

Patients with endometrial cancer who were morbidly obese tended to have worse OS and DFS compared with women who were obese (BMI <40 kg/m^2^). BMI >40 kg/m^2^ was determined to be an independent prognostic factor for both OS and DFS. Stage and comorbidities were also detected as independent prognosticators for OS. Keeping in mind that comorbidities and BMI are modifiable factors, efforts in this population should be focused on medical optimization and lifestyle alterations, particularly kg loss.

## Figures and Tables

**Table 1:**
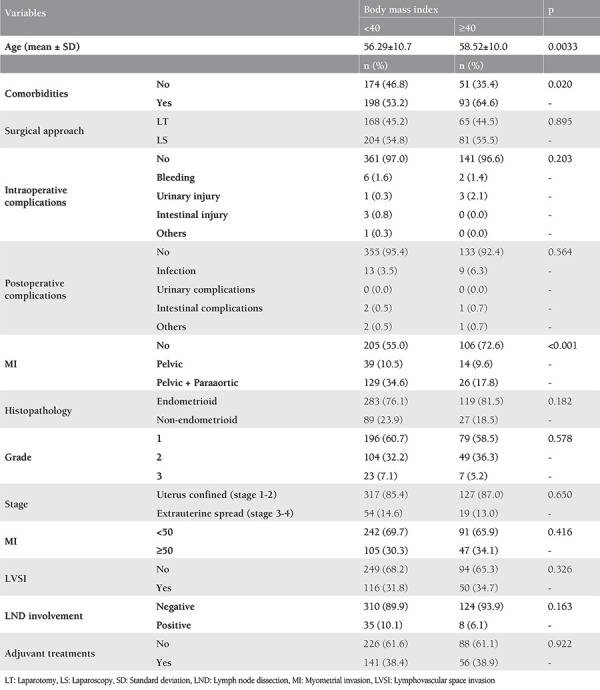
Patients’ characteristics

**Table 2:**
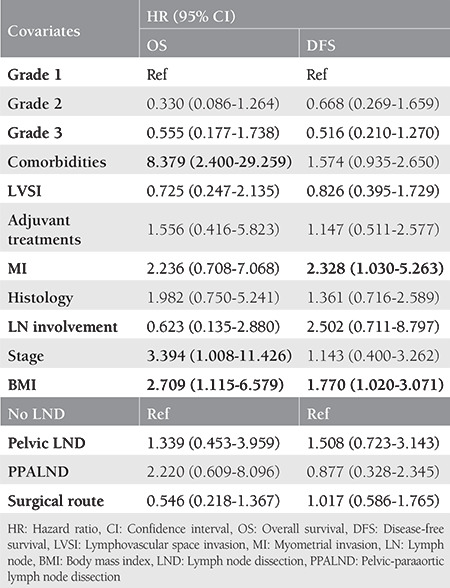
Multivariate analysis of age adjusted overall survival and disease-free survival
